# Undifferentiated Altered Mental Status: A Late Presentation of Toxic Acetaminophen Ingestion

**DOI:** 10.1155/2012/162387

**Published:** 2012-06-28

**Authors:** Thomas E. Robey, Edward R. Melnick

**Affiliations:** Department of Emergency Medicine, Yale University, 464 Congress Avenue, Steuite 260, New Haven, CT 06519-1315, USA

## Abstract

Altered mental status is a common undifferentiated presentation in the emergency department. We describe a case of acetaminophen-induced acute liver failure that was diagnosed and treated prior to obtaining definitive historical or laboratory information about the etiology. The physical exam finding of scleral icterus in this case was a key element to rapid identification and treatment of this life-threatening condition. A discussion of appropriate N-acetylcysteine treatment for acute liver failure and acetaminophen intoxication is included.

## 1. Introduction

The etiology of altered mental status can lie among a wide range of causes, most commonly, neurologic, toxicologic, infectious, traumatic, metabolic, and psychiatric [1, 2]. When patient history is unavailable, each of these possibilities must be entertained. In critically ill patients, treatment decisions must be made in advance of definitive diagnosis, either empirically or based on the best data available at the time. We describe a case of acute liver failure in the context of a late presentation of acetaminophen toxicity that was treated with N-acetylcysteine (NAC).

## 2. Case Presentation

A 61-year-old woman was found confused on her floor by paramedics after they were called by concerned neighbors, who had not seen her for five days. Upon arrival to the emergency department (ED), the medics reported seeing no unusual circumstances in her residence but relayed that she had been “acting odd” at a neighborhood party five days prior. The patient was unable to provide any information secondary to her altered mental status. Initial vital signs in the ED were a temperature of 36.6°C, a pulse of 94/min, respiratory rate of 18/min, a blood pressure of 134/59, and O_2_ saturation of 96% on 4 L O_2_ by nasal cannula placed in the field. Medications found in the home by EMS included rosuvastatin, nadolol, albuterol inhaler, citalopram, and a lidocaine patch. The above medications for a medical history of hypertension, hyperlipidemia, asthma, bipolar disease, and chronic back pain were confirmed by telephone with her daughter. She had a partial gastrectomy for a peptic ulcer eight years prior and had been sober from alcohol for five years. Physical exam revealed a lethargic uncooperative patient with incoherent verbalization who withdrew only the right upper extremity from noxious stimuli. She had icteric sclera, mild jaundice, and dry mucus membranes. There were no topical medication patches in place. The stool was negative for occult blood, and chest and abdominal exam were unremarkable. There was no nystagmus, and the remainder of her neurologic exam was limited by her inability to participate. 

Suspecting neurological, metabolic, or toxicologic catastrophes, a CT-head and full complement of laboratory tests were ordered. The CT-head showed no acute abnormality. Hematologic studies revealed a white blood cell count of 3,600/mm^3^, a platelet count of 138,000/mm^3^, and a hemoglobin level of 15.5 g/dL. Electrolytes were normal except for a blood urea nitrogen (BUN) of 53 mg/dL (normal 7–20 mg/dL). Her initial creatinine level was 0.7 mg/dL, and her GFR was 90. Other lab results included a lactate of 2.7 mmol/L (0.4–2.2 mmol/L), an INR of 3.07, a troponin T of 0.04 ng/mL (normal <0.01 ng/mL), and a creatine kinase of 531 U/L (24–170 U/L). Serum toxicology levels were normal, with acetone present but less than 10, and an acetaminophen (APAP) level of 3 mg/L (10–20 mg/L). Transaminase levels were initially delayed and returned one hour later with elevation of the aspartate aminotransferase (AST) level up to 1240 U/L (0–34 U/L), alanine aminotransferase (ALT) to 1450 U/L (0–34 U/L), and alkaline phosphatase (AP) of 238 U/L (30–130 U/L). Potassium and ammonia levels were unavailable due to hemolyzed blood samples. Direct and indirect bilirubin levels were 5.8 mg/dL (<0.2 mg/dL) and 9.3 mg/dL (<1.2 mg/dL), respectively. Urinalysis was dark with a specific gravity of 1.018, moderate bilirubin, moderate ketones, and no evidence of infection. The electrocardiogram was normal except for a premature atrial complex. A chest X-ray was normal. No prior records were available for comparison.

The patient was given 2 L of normal saline based on a clinical exam consistent with dehydration. Intravenous NAC treatment was initiated to treat acute liver failure. The normal APAP level did not contradict the NAC use, but pointed to a possible etiology for this patient's liver failure. She was admitted to the intensive care unit and evaluated by the liver transplant team. An ammonia level drawn 12 hours after presentation was 70 *μ*mol/L (11–35 *μ*mol/L), and she demonstrated asterixis as her mental status cleared. A complete 20-hour IV NAC regimen was completed, and lactulose given PO, and the patient recovered without serious sequelae. An ultrasound of the liver showed minimal evidence of fatty infiltration but revealed no sonographic evidence of cirrhosis. At discharge five days later, her AST had decreased to 96 U/L, her ALT to 212 U/L, and her AP to 174 U/L. Her bilirubin levels remained high at direct of 13.3 mg/dL and total of 17.7 mg/dL, but the INR had normalized to 1.27. After return of mental capacity, the patient denied relapse of her sobriety from alcohol and endorsed taking “a lot of Tylenol” four days prior to presentation. She refused to comment on the specific quantity or her motivation for taking the pills.

## 3. Discussion

Late presentations of acetaminophen ingestion may be difficult to identify when patient histories are unavailable. This case is an excellent illustration of how the glutathione precursor, N-acetylcysteine, is the appropriate treatment for acute liver failure with or without an elevated initial APAP level. After the altered mental status resolved, subsequent history confirmed the patient's APAP use, and physical exam and laboratory tests demonstrated hepatic encephalopathy. The patient's mental status cleared with NAC therapy, which treated APAP toxicity and the fulminant liver failure. The Rumack-Matthew nomogram is of limited use in this case, owing to the uncertain time of ingestion, the possibility of multiple ingestion events, the delayed presentation, and the low APAP level [3]. A distant history of alcohol abuse along with a trace of fatty liver could have left her more susceptible to the effects of APAP toxicity, but it is more likely that she consumed a large number of pills and, owing to her developing encephalopathy, was not able to keep track of the quantity. 

This patient's grade III (incomprehensible speech and arousable to stimuli) encephalopathy predicts a 40–50% recovery [4] without treatment. However, patients with signs and symptoms of hepatic injury and any suspicion of APAP ingestion should be immediately started on NAC [5]. There are several pathways by which NAC is antidotal to APAP's metabolite, N-acetyl-p-benzoquinone imine (NAPQI). In addition to preventing toxicity via decreasing NAPQI formation [6], NAC increases the ability to detoxify NAPQI [7] and treats liver toxicity through nonspecific effects [8, 9] of increased tissue oxygenation and reduction of tissue free radical formation. These nonspecific mechanisms are the likely reasons for clinical recovery in this case. 

The nonspecific hepatoprotection offered by NAC has been proposed to be superior with intravenous delivery [9, 10]; PO formulations may prove effective for fulminant liver failure but have not been studied to date. In cases such as this when the patient's minimal alertness increases risk of aspiration, IV NAC administration should be considered over PO formulations. IV NAC yields equivalent outcomes as the PO formulation, and, although it is more expensive, it is easier to administer (due to the foul taste and multiple doses of the PO formulation) and requires a shorter time course, which ultimately leads to cost savings for the hospital [11]. 
